# Finding new purpose for vacancies in rural areas: a taxonomy of coworking space business models

**DOI:** 10.1007/s11365-023-00867-0

**Published:** 2023-05-23

**Authors:** Nina Thornton, Martin Engert, Andreas Hein, Helmut Krcmar

**Affiliations:** KrcmarLab, School of Computation, Information and Technology, Technical University of Munich, Boltzmannstrasse 3, Garching, 85748 Germany

**Keywords:** Coworking Space, Rural Area, Business Model, Business Model Canvas, Entrepreneurship

## Abstract

As a result of the rural exodus over the last decades, unused vacancies in rural areas are at risk of falling into disrepair. Given the current trends of flexible workplaces and people returning to rural areas, their repurposing as coworking spaces (CWSs) by entrepreneurs poses a potential for sustainable future-oriented workplace solutions. However, there is little to no guidance on the structural configuration and business models of CWSs in rural areas available for these entrepreneurs. We apply a structured empirical research approach to create a comprehensive and specialized taxonomy, including a literature review and eleven interviews with operators of rural CWSs in Germany. The resulting taxonomy of business models of CWSs in rural areas based on an extension of the business model canvas contributes to the knowledge base on rural CWSs. We evaluate its usability through a case study and an entrepreneurial operator of a rural CWS, underlining its entrepreneurial and practice-oriented purpose. The study addresses several urgent topics, such as the future of work and new work (places), which enable and accelerate the development of CWSs outside agglomerations consequential to the COVID-19 pandemic. It also promotes social and sustainable entrepreneurship and the revitalizing, enhancing, and increasing of digital accessibility of rural regions.

## Introduction

Coworking spaces (CWSs) embody novel concepts of the new workplace and meet the need for flexible but professionally equipped workplaces. They offer an alternative to working from home or commuting long distances to work and have emerged rapidly in the past decade (Leclercq-Vandelannoitte & Isaac, 2016; Waters-Lynch & Potts, 2017).

Even though CWSs emerged and are mainly attributed to urban areas (Waters-Lynch & Potts, 2017), we observe a shift towards establishing CWSs in rural areas. This trend relates to the *“work from anywhere”* (Choudhury, 2020) approach. Large corporations such as Siemens, Facebook, and Twitter have adopted and promoted this practice in response to the COVID-19 pandemic (Choudhury, 2020). Additionally, people are increasingly moving from cities to rural areas since the pandemic, as exemplary data from the USA and Germany show (Dähner et al., 2019; Merten, 2020; Rose, 2020). The often unique environmental settings in remote and rural areas provide opportunities for diversification of service offerings compared to their urban counterparts, including, for instance, recreational activities. Furthermore, CWSs in rural areas can revitalize rural communities, particularly when set up in vacancies (Engstler & Mörgenthaler, 2018).

However, little is known about their implementation, including structural composition, economic feasibility, and profitability (Fuzi, 2015). It is uncertain whether franchise-based, one-size-fits-all business models of CWSs in urban settings can and should be applied unaltered to rural CWSs (Bähr et al., 2020). Their environmental and demographic circumstances, including rurality, seclusion, or identity-creating character (Voll et al., 2021), are far more diverse and individual than urban CWSs (Bähr et al., 2020). In addition, it takes more than mere emergence and existence of CWSs in peripheral areas to utilize the existing potential and achieve goals such as reviving vacancies (Voll et al., 2021). Lastly, existing rural CWSs tend to be microenterprises created and operated by highly motivated but relatively inexperienced entrepreneurs with few resources available for trial ventures (Bähr et al., 2020). Providing specific guidance on how to configure business models of rural CWSs can help entrepreneurs to build sustainable businesses together with local communities. Therefore, we strive to answer the following research question: *What elements constitute business models of coworking spaces in rural areas?*

We conduct a literature review on CWSs and qualitative expert interviews with operators of rural CWSs in Germany to develop a taxonomy. We draw on proven design ideas from business model research and apply them to the specific case of rural CWS. The resulting taxonomy describes the characteristics of business models for rural CWSs we identify. We test and evaluate its usability through a case study of a planned CWS in a rural community in Germany.

Our results guide founders and policymakers on how to facilitate the opening and successful implementation or restructuring of rural CWSs. We also intend to motivate managers of CWSs and other enterprises to consider entering this promising entrepreneurial sector. Moreover, we contribute to research on CWSs by providing an analytical perspective on and possible manifestations of rural CWS business models. More successful rural CWSs would contribute to sustainability by reusing resources and strengthening rural regions. Therefore, this study’s contribution has economic and societal relevance.

## Research methods

### Literature review

CWSs are a promising and novel entrepreneurial phenomenon (Seo et al., 2017). Since it is an emerging topic, there is little and heterogeneous research literature on CWSs in general and CWS in rural areas (Josef, 2017; Seo et al., 2017). Given the inconsistent use of the term CWS and the multitude of different perspectives taken, we conducted a comprehensive literature review to clarify and organize the subject.^1^ Overall, we found a strong focus on empirical studies on CWSs compared to a smaller number of conceptional publications. There also is a surplus of literature concerned with CWSs in urban areas as opposed to rural CWSs.

From the literature review on CWSs both in urban and rural contexts, we identified a lack of rigorous and comprehensive classification. Although some taxonomies of rural CWSs exist (BMEL, 2021; Bähr et al., 2020; Voll et al., 2021), their dimensions are either not clearly defined or too complex to enable comparability. Two approaches exist to create a structural categorization, differing in their development methodology. A *“typology is conceptional while a taxonomy is empirical”* (Bailey, 1994, p. 6). Based on the heterogeneous nature of the research field of CWS and the limited availability of conceptual knowledge on rural CWSs, we decided to develop a taxonomy following Nickerson et al. (2013).

### Taxonomy generation: qualitative interviews

The purpose of this taxonomy is the general distinction between CWSs in rural areas from an economic point of view. Therefore, we chose business models as a meta-characteristic, *“serv[ing] as the basis for the choice of characteristics in the taxonomy”* (Nickerson et al., 2013, p. 343). We selected the Business Model Canvas (BMC) by Osterwalder and Pigneur (2010) to ensure comparability and generality. The BMC is recognized by both theorists and practitioners, especially in management and entrepreneurship (Salwin et al., 2022). It has a clear structure and captures an entire business model in nine components. Key Partners, Key Activities, Key Resources, and Cost Structure are efficiency-focused. Customer Relationships, Channels, Customer Segments, and Revenue Streams are value-centered. They all group around the Value Propositions component (Osterwalder & Pigneur, 2010).

We conducted qualitative interviews to develop and elaborate the taxonomy. We held eleven of the overall 16 interviews from the end of November 2020 to the beginning of March 2021. We used the geographical positioning in a rural municipality as a criterion for the interviewee selection. The official definition of spatial classification by the German Federal Institute for Research on Building, Urban Affairs, and Spatial Development served as our reference. CWSs in towns with over 20,000 inhabitants were excluded. We interviewed operators of CWSs in rural areas of Germany (CWS1 to CWS11) and utilized the outcomes for the taxonomy design. They were set up as expert interviews, as the operators belong to the taxonomy's aspired user and recipients group. We conducted semi-structured interviews and adjusted the pool of questions as new insights emerged during the process. An overview of the most pertinent questions is included in Appendix B.1.

After the interview conduction and transcription, we applied qualitative content analysis to extract and prepare relevant interview data to elaborate the proposed taxonomy. We thereby created an information base that exists detached from the original texts (Gläser & Laudel, 2010, p. 200) by transferring statements from the interviews into a previously created search grid. The structure of the standardized search grid we developed and applied can be found in Appendix B.2.

From the qualitative interviews, we followed an empirical-to-conceptional approach (Nickerson et al., 2013) to choose the characteristics and dimensions of the taxonomy, using the BMC structure and its triple-layered extension presented by Joyce and Paquin (2016). This model includes elements that exceed a company’s traditional value creation process reflected in the conventional BMC by adding two layers. One is dedicated to disaggregating the business model regarding social aspects, whereas the other itemizes its environmental impacts.

The initial taxonomy resulting from this first iteration included 18 dimensions and between two and seven manifestations in each dimension, capturing the heterogeneity of business models in CWS in rural areas. In the next step, we evaluated and refined the taxonomy.

### Taxonomy evaluation: case study and expert interview

We applied a case study as an empirical evaluation method to prove the usability of the taxonomy in a practical manner. Consequently, we used our taxonomy to create a business model for a CWS in a real-life rural environment. The selected site is located outside an agglomeration, and it classifies as a rural municipality due to its population of less than 5,000 (as of 2019).

The case study consists of multiple sources of evidence, namely documentation, archival records, interviews, direct observations, participant observation, and physical artifacts (Yin, 2018, pp. 126–127). We conducted six interviews (KR1 to KR6), listed in Appendix B.3.

Contrary to the interviews with operators of rural CWSs, we used no widely standardized interview guideline questions. Instead, we compiled an individual set of questions before conducting each interview, which addressed relevant topics for the respective interviewee. Lastly, we obtained direct observations at an on-site visit to the premises of the chosen location.

In addition to the case study, we evaluated the taxonomy with an entrepreneurial operator of a rural CWS who had been in the planning phase at the time of the interview. We sent out the taxonomy before the interview for the entrepreneur to apply it themselves. This interview further underlined that the taxonomy developed is helpful for practitioners because it presents the various options for business model design in a structured and concrete way.

## Results

### Coworking spaces from an entrepreneurial perspective

A CWS’s general offering can be best summarized as an office-as-a-service or described as an *“’ on-demand office facility”* (Blagoev et al., 2019, p. 895). CWSs often provide a more comprehensive range of services, such as regularly organizing events and workshops, coaching, mentoring, or handling administrative tasks for users (Bouncken et al., 2020b). A CWS is further characterized by a lack of direct goal-monitoring or task management by other employees or supervisors (Bouncken et al., 2020a, b, c). This gives the user autonomy and comes alongside the reduction or absence of organizational hierarchy (Bouncken, Kraus, et al., 2020a, b, c). Instead, community and mutual trust are built between autonomous users, replacing hierarchical structures (Bouncken & Reuschl, 2018). Typical customers of CWSs are *“freelancers, new start-ups and graduates”* (Bednár & Danko, 2020, p. 114) and seem to benefit from these characteristics (Bouncken & Reuschl, 2018; Bouncken et al., 2018; Bouncken et al., 2020a, b, c; Fuzi, 2015).

Rather than focusing on how CWSs enable entrepreneurship, we will assess them as entrepreneurial entities. *“Entrepreneurs [generally] depend on business opportunities to seek value-creation”* (Hummels & Argyrou, 2021, p. 4). The rapid emergence of CWSs in recent years showcases plenty of opportunities for this form of entrepreneurship.

### Typologies of coworking spaces

*“The growth of coworking spaces has led to the[ir] diversification”* (Kraus et al., 2022, p. 8), encouraging researchers to classify and organize different forms and variants. Various researchers have developed categorizations of CWSs, a selection of which is presented in Table 1.

**Table 1:** General Typologies of Coworking Spaces and their Distinguishing Features

Document	Typology/ Taxonomy	Distinguishing Features		
		Value Added	Governance	Openness
Avdikos and Merkel (2020)	Entrepreneurial-led Coworking Spaces	x	x	
	Community-led Workspaces			
Bouncken et al. (2018)	Corporate Coworking-Space	x	x	x
	Open Corporate Coworking-Space			
	Consultancy Coworking-Space			
	Independent Coworking-Space			
Bouncken et al. (2020b)	Efficiency-centered business model	x	x	
	User-centered business model			
	Development-centered business model			
	Platform business model			
Gandini and Cossu (2021)	Neo-Corporate Model	x	x	
	Resilient Model			
Ivaldi et al. (2020)	Infrastructure Coworking	x		
	Relational Coworking			
	Network Coworking			
	Welfare Coworking			
Kojo and Nenonen (2016)	Public Office	x		x
	Collaboration hubs			
	Incubators			
	Third Places			
	Co-working Hotels			
	Shared Studios			
Marchegiani and Arcese (2018)	Vertical Coworking Spaces			x
	Horizontal Coworking Spaces			
	Niche Coworking Spaces			
Spinuzzi (2012)	Community Work Space	x		
	Unoffice			
	Federated Space			
Yang et al. (2019)	Revenue Model	x		
	Synergistic Model			
	Customer Contact Model			
Zinke et al. (2018)	Corporate operators		x	
	Non-Corporate operators (Type 1, Type 2, Type 3)			
	Public operators			

We identified three rough distinguishing features for the typologies described in the papers. First, most authors use the (anticipated) type of added value of the CWS to categorize models. Second, some employ the kind of operator (governance) as a distinguishing feature, and third, the openness to different user groups. However, the typologies reviewed exist independently and are not related to each other beyond these broad distinctions. Our examination thus primarily points to various influences determining a CWS business model.

Most of the studies refer to CWSs in urban contexts. The underlying observation is that *“Coworking spaces are often set up in central, exposed, and attractive locations, matching an attractive interior to the external urban space”* (Bouncken, Kraus, & Martínez-Pérez, 2020a, b, c, p. 1467). Though this is true, there has recently been a notable trend toward CWSs in more rural areas (Bouncken, Kraus, & Martínez-Pérez, 2020a, b, c, p. 1476). The recognized potential of CWSs in rural areas mainly drives this trend shift. However, little is known about the particularities of rural CWSs and their business models.

### Introducing rural coworking spaces

We found limited typologies specific to rural CWSs or CWSs in rural and peripheral locations (Voll et al., 2021). We present these in Table 2, as the distinguishing features found are far more diverse and incomparable with those derived from the typologies presented in Table 1.

**Table 2:** Typologies of rural Coworking Spaces and their Distinguishing Features

Document	Typology	Distinguishing Features
BMEL (2021), Bähr et al. (2020)	Classical coworking Commuter port Bottom Hub Retreat Workation Integrated housing and work projects New village center	Place, Spread, Resilience, Community, Intake, Region
Voll et al. (2021)	Independent individual operators of CWS Cooperatives, collectives, and teams focus on one place Municipal CWS (e.g., docked with local economic development agency) Scaling models, networks (if applicable regional focus or also CWS promoted by companies) Temporary formats No coworking	Form of incorporation
Voll et al. (2021)	Classical coworking Mixed-use cases: mainly services Mixed-use cases: mainly public benefit-oriented Coworkation Coliving Spaces CWS as a complement to, or spin-off from, existing urban CWS	Not explicitly defined

The variety suggests a greater diversification of CWSs in rural than urban areas (Bähr et al., 2020). It complicates the comparison of the different typologies – both with one another and with those observed for urban CWSs.

The large number of categories, the hybrid forms, and the resulting unclear boundaries between the identified types raise the question of whether defining general (arch)types for CWSs in rural areas is beneficial or whether we can circumvent that without a loss of meaningful contribution.

### Particularities of rural coworking spaces

Furthermore, the question arises as to what circumstances cause the diversity of CWSs in rural areas. The first explanation is the **environmental variety** in rural areas which differs from urban regions (Ferreira et al., 2015). Urban areas display uniform features, such as a high population density and a vivid entrepreneurial scene (Cabral & Van Winden, 2020; Nakano et al., 2020). In contrast, rural areas influence the BM of a CWS more strongly due to their identity-forming character and surrounding features (Voll et al., 2021), such as a forest or a waterside. This explains the variety of entrepreneurial activities outside agglomerations (Roundy, 2019) and the **diverse clientele** observed in rural CWS establishments (Bähr et al., 2020). It also suggests the importance of taking advantage of these unique environmental factors. As entrepreneurship and research on entrepreneurship concern efforts to understand how to discover, exploit, and create entrepreneurial opportunities (Hummels & Argyrou, 2021), we focus on this aspect.

The utilization of available features and resources also aligns with research on **frugality and sustainability** or green entrepreneurship that is presently being given considerable attention (Gregori & Holzmann, 2020; le Loarne et al., 2022). The primary goals of frugal entrepreneurship are fulfilling a greater good for society and contributing to sustainability through its offerings (Hossain & Sarkar, 2021). *“[H]istoric sacred places match well with coworking”* (Wright, 2018, p. 57), as they often inherit features from their previous usage that *“meet the needs of coworking spaces such as having access to transit, being near amenities and housing, and consisting of a variety of interior spaces”* (Wright, 2018, p. 57). If remaining vacant, these buildings often have to be demolished (Dähner et al., 2019).

Countering vacancies in rural communities through CWSs can ensure sustainable **reuse and revitalization** of entire town centers (Engstler & Mörgenthaler, 2018, p. 23). In addition, integrating rural CWSs into the premises of the administration of municipalities can bring people and further ideas for innovation and digitalization into rural regions (Prochazka & Wingartz, 2019). Rural CWSs can also become connection points to more urban areas and provide opportunities for urban–rural collaboration (Avdikos & Merkel, 2020). Moreover, as the services provided by CWS in more remote and less populated areas are typically more pluralistic (Marchegiani & Arcese, 2018), they may also function as social infrastructures (Avdikos & Merkel, 2020). In summary, CWSs can be *“very much seen as bringers of new opportunities for rural areas”* (Mediteranean, 2018). Because of that, CWSs may also benefit from market and community-based strategies supported by community members of small towns and rural areas to promote entrepreneurship (Roundy, 2019).

### Elaboration of the taxonomy of business models of coworking spaces in rural areas

We selected the BMC as an underlying structure to choose the taxonomies’ characteristic and extended it with the triple-layered BMC by Joyce and Paquin (2016). To illustrate our taxonomy and integrate all components in an aggregated structure, we adopted the form of a morphological box (Table 3).

The different manifestations of the characteristics are not mutually exclusive, contrary to the general taxonomy definition (Nickerson et al., 2013) and the typical application of a morphological box. Implementing and including different manifestations within a type (e.g., Value Proposition Type) and combining them in a business model seems advantageous. This was evident from all interviews conducted, even in cases focusing on specific features.

The following section explains the individual segments of our taxonomy. They represent the main findings of this study. We exclude the cost structure component of the BMC from the overall presentation due to its uniqueness and only describe it briefly. Setting up the cost structure after applying the presented taxonomy to fit best, including all environmental factors, is recommended.

**Table 3:** Taxonomy for Business Models of Rural Coworking Spaces

Business Model Component	Categorization	Characteristics						
Customer Segment Type	Dependency	Organization-dependent			Independent			
	Main Motivation	*Situational*			Business Related Need		Personal Preference	
	Temporal Commitment	Long- and Medium-Term		Short-Term	Long- and Medium-Term	Short-Term	*Situational*	
Value Proposition Type	Value Offering	Spatial Platform		Office-as-a-service			Inspirational place to work from	
	Implementation	Exchange (Facilitation) Platform	Presentation Platform	Workplace-as-a-service	Fixed Office Space	Workation/ Co-Living	Space Design	Exterior Environment
Channel Type	Ownership	Self-Owned				Partner-Owned		
	Addressing	Individualized		Standardized		Standardized		
Customer Relationship Type		Tenancy	Service Relationship	Membership	Community	Co-Operation	Co-Creation	
Revenue Stream Type	Revenue Model	Renting		Subscription Plan(s)		Pay-as-you-go		
	Pricing	Feature-based		Fixed		Negotiation		
Key Resource Type	Resource Category	Site Factors			Functional Infrastructure	Social Resources		
	Specification	Premises		Venue	Situational	Community	Network	
Key Activity Type		Onboarding and Networking		Community Management		Marketing and Awareness-Raising		
Key Partnership Type		Strategic Alliances		Fusion		Coopetition		
Governance Type	Corporate Form	Sole Proprietorship		Limited Liability Company	Entrepreneurial Company		Cooperative	
	Activity Prio	Main			Secondary			
Community Type		Municipalities		(Local) Companies		Local Residents		
Type of Scale of Outreach		Close (Rural) Surroundings		Intermediate Distance		Wide Distance		

#### Customer segments of rural coworking spaces

We identified various characteristics dividing the clients of rural CWSs into different segments. For example, organizational affiliation, motivation, and time commitment to the CWS serve as distinguishing criteria. For **Organization-Dependent Long- and Medium-Term** customers, the rural CWS either complements or replaces the permanent workplace in the long and medium term. The clients from this customer segment are either individual customers or companies. **Organization-Dependent Short-Term** customers are also affiliated with a company or institution. They are working groups or boards of directors that purchase the offerings of the rural CWS for specific events, such as seminars, workshops, or meetings.

**Independent Business-Related Need Long and Medium-Term** customers are self-employed and micro-enterprises that permanently or partially locate their workplace in the rural CWS. **Independent Business-Related Need Short-Term** customers are not businesses with a fixed location but independent service providers who carry out their work activities in the rural CWS. **Personal Preference** customers visit or use the rural CWS for individual and non-business reasons. This customer type includes students, doctoral candidates, workationists, or event visitors.

#### Value propositions of rural coworking spaces

We identified three overarching value proposition categories for rural CWSs. These offer a spatial platform, an office-as-a-service, or an inspiring workplace. Each subdivides into two to three subcategories. An **Exchange Facilitation Platform** is realized by providing spaces specifically designed for (social) exchange, sometimes accompanied by community management and organization and the holding of events. In **(Re-)Presentation Platforms**, different clients can present and introduce their projects, products, or services to a self-defined or more coincidentally emerging audience in the rural CWS.

**Workplace-as-a-service** refers to offering individual workstations in freely selectable or permanently assigned desks on a large free area. The equipment at the desks and in the space may differ from case to case. **Fixed Office Spaces** are office rooms that function as individual offices, team rooms, or shared offices. This can also include virtual offices, which include providing a business address and accepting and forwarding mail or packages. **Workation** or **Co-Living** typically consist of offers for holiday establishments combined with those associated with providing a workplace. This includes, in particular overnight accommodation, leisure activities, or the provision of meals.

The installation of plants or the focus on an appealing interior design and furnishings are implementations to support creating an inspiring working environment. A quality standard or a specific corporate image can add to the **Space Design**. The value proposition **External Environment** is particularly realized when the CWS is located in a natural environment, e.g., near a lake, the sea, a forest, or a remarkably tranquil place in a village or small town.

#### Channels of rural coworking spaces

**Individualized Self-Owned** channels are predominantly applied to raise awareness and marketing, as they allow for direct peer-to-peer communication. In addition, the individualization of conveyed information via telephone calls, face-to-face communication, or personal emails and messages makes their usage highly effective.With **Standardized Self-Owned** channels, the generation of messages or emails becomes automated, and services are booked directly via the website. As a result, reliable and straightforward booking software can be crucial, particularly when offering overnight stays or operating at a fairly remote location with a geographically distant customer base. **Standardized Partner-Owned** channels depend mainly on the key partners of the respective rural CWS business model implementation. Possibilities include the presence on web-based nationwide platforms for rural CWSs, display in other rural and urban CWSs, marketing channels of the respective community, business or educational institutions, local newspapers, radio, and TV stations.

#### Customer relationships of rural coworking spaces

The customer relationships we observed in rural CWSs cover a wide range, from more traditional separated provider-recipient models to deeply entangled professional and social relationships. This depends on the selection and use of channels and the offered services and governance in place.

A **Tenancy** primarily occurs in the context of long-time office leasing. A **Service Relationship** refers to customers using the premises as a workplace, meeting location, or accommodation for a limited time. A **Membership** offers the customer various options, such as access to events, information, or multiple rural CWSs in an existing network. A software-based implementation enables booking different specifications of offered value propositions.

The **Community** customer relationship is difficult to measure or grasp. It emerges from personal contacts and business and is increasingly encouraged by numerous companies. When the governance is realized as a cooperative (as a form of company), customers take on the role of shareholders of the rural CWS. Users then might operate the rural CWS themselves, resulting in **Co-Operation**. In the case of **Co-Creation**, customers do not run the CWS but become a crucial part of the creation and (further) development of new value.

#### Revenue streams of rural coworking spaces

We categorize two defining aspects of revenue streams: the revenue model and the pricing mechanism. Possible revenue models include **Renting**, which represents the sale of the exclusive right to use (parts of) the rural CWS for a specific time. **Subscription Plans** enable continuous access to a service, and **Pay-as-you-go** entails the sale of time-limited usage quotas. As for pricing, it depends on access to different service levels in **Feature-Based** pricing, pre-determined prices for various services in **Fixed** pricing, or individual cases in the case of **Negotiation**.

#### Key resources of rural coworking spaces

We established an overarching three-part categorization into external circumstances (site factors), internal features (functional infrastructure), and human-related resources (social resources). The **Premises** in which the CWS is allocated and its structure largely determine the form of the other business model components. For rural CWSs, the **Venue** and its location are also crucial resources and can be advantageous in two respects. It is either located close to nature and thus of touristic importance or logistically advantageous – for locals or more remote users. The functional **Internal Infrastructure**, such as secure and robust internet connectivity, is also essential, as it can compete with or exceed the locally prevailing standard. The interior can also reflect the business model by integrating upcycled pieces to convey a focus on sustainability.

The user **Community** generally contributes to the offered and proposed value propositions and is particularly vital in the context of the described customer relationship, co-operation, and co-creation. A **Network** includes (local) connections and the know-how that emerges from or is only accessible through an exchange.

#### Key activities of rural coworking spaces

**Marketing and Awareness-Raising** typically involve activities for new and not yet established ventures and thus also occur in rural CWSs. They are additionally continuously integrated into the business concept through the organization of events open to the public and the overall feeding of the channels. **Onboarding and Networking** include the establishment and maintenance of key partnerships. The specific activity characteristics of this type might differ considerably. Examples include onboarding other rural CWSs to integrate into the business model or networking with companies in the nearest larger cities to establish collaborative relationships. **Coworking Management** is a set of activities concerning the internal operations of the rural CWS. It involves the maintenance of the space and the various implemented customer relationships.

#### Key partnerships of rural coworking spaces

**Strategic Alliances** are beneficial partnerships with non-competitors. They are, for instance, formed with the respective municipalities, exemplarily for marketing purposes. **Coopetition** arises between competitors and, thus, between different companies from the same sector. However, the extent of cooperation and mutual support seems characteristic of rural CWSs. One example is a national cooperative for rural coworking spaces, of which most interviewees were members. **Fusion** goes beyond cooperation and represents the closest relationship between rural CWSs observed. It entails integrating other CWSs into the own business model and thus merging with potential competitors.

#### Governance of rural coworking spaces

We included the following three components from the social stakeholder layer of the triple layered BMC. We describe Governance in two parts: the **Corporate Form** and **Activity Prioritization**. Most corporate forms we found within rural CWSs are relatively simple, which can be explained by their small size and rather early stages of development. We observed **Sole Proprietorships**, **Limited Liability Companies**, and a singular occurrence of a more complex **Entrepreneurial Company** and a **Cooperative**.

**Activity Prioritization** refers to the operation and governance of each CWS business model. More than half of the interviewees stated that its operation is their **Secondary** occupation. Though, mostly the respective **Main** career is related to the CWS in some form.

#### Involvement of local communities in rural coworking spaces

**Municipalities** can be involved as political entities or impact rural CWSs as geographical units. In both cases, CWSs often pose substantial benefits, exemplarily by using vacancies and revitalizing immediate surroundings. Additionally, the CWS might indirectly serve the municipality’s development as a business location. However, from our interviews, we have found the participation of the political institutions within the municipalities has been minimal so far. **(Local) Companies** benefit, for example, from providing premises for periodic use or – in the case of gastronomy – by providing daily catering for the CWSs. The **Local Residents**, not part of the user group, profit from and contribute to the integration of local communities. CWSs enable this by providing a meeting place for all residents or creating a central point for club leaders, clubs, and engaged people.

#### Scale of outreach of rural coworking spaces

The observed types of the scale of outreach are **Close (rural) Surroundings**, exemplarily represented by the commuters, which typically have their residency in the immediate surrounding areas of the rural CWS, **Intermediate Distance,** and **Wide Distance**.

#### Cost structure of rural coworking spaces

From the interviews, we discovered the individuality of cost distribution. We identified some recurrent categories: Rent, **Personnel Costs**, **Booking and Accounting Software**, **Infrastructure**, and **Additional Costs**. The most significant difference in costs relates to whether there are employees in the rural CWS. If this is the case, personnel costs make up the largest share of the costs, otherwise, it is the rent. All other cost distributions depend on different business model components and their realization.

## Evaluation of the taxonomy

As described in "Taxonomy evaluation: case study and expert interview" section, we conducted a case study using our taxonomy in the concrete context of a historic building in a real municipality. The proposed selection of implementations is based on selected sources, location factors, and information concerning the different dimensions of the taxonomy. This instantiation of a rural CWS business model based on the morphological box is presented in Table 4.

**Table 4:** Exemplary Business Model Composition Proposal for a Rural Coworking Space

Component	Chosen or Given Characteristics	Reasoning and justification
Customer Segment Type	Organization-Dependent Long and Medium-Term	We identified a large number of employees in the municipality
	Organization-Dependent Short-Term	Some IT companies also exist in the area that could use the space as a facility for events
	Independent Personally Motivated	Due to the city's proximity, tourists can use the CWS
Value Proposition Type	Exchange Platform	A community meeting place is politically encouraged
	(Re-)Presentation Platform	Well-equipped companies in the area could benefit from an external presentation platform
	Workplace-as-a-service	Employees are eager for an alternative to their home office
	Space Design	The open spaces of the venue offer a welcome opportunity for high-quality furnishings
Channel Type	Indirect Self-Owned	Due to the high costs, the focus should be on low-maintenance automated channels
	Direct Self-Owned	A website and a regular newsletter would be advisable
	Partner-Owned	Local press, in particular, is seen as useful
Customer Relationship Type	Service Relationship	A micro-study regarding the number of working days indicates the potential for a service relationship model
	Membership	Memberships should generate regular income from the many employees
	Community	Depending on the type of Governance, establishing a community might be feasible
	Co-Operation	The implementation of co-operation also depends on the type of governance
Key Resource Type	Premises	The building is of historic importance and provides a suitable architectural structure
	Venue	Its location is in the center of the municipality, which is in the suburbs of a large city
	Functional Infrastructure	A fiber optic connection can be provided
	Network	In addition to political interests, there is a foundation for connections to surrounding businesses
Key Activity Type	Community Management	For the day-to-day operation, a form of community management is recommended
	Marketing and Awareness-raising	As the concept of CWSs is relatively unknown locally, awareness-raising is essential
Key Partnership Type	Strategic Alliance	The network in place constitutes a valuable basis for developing strategic partnerships
	Coopetition	Joining the cooperative for rural CWSs in Germany is a promising activity
Governance Type (Activity Prioritization)	Limited Liability Company (Secondary)	If a suitable entrepreneur can be found, a limited liability company’s simple but profitable form is recommended
	Cooperative (Primary)	If several interested parties find themselves collaborating, it is advisable to set up a cooperative
Local Community Type	Municipality	Due to apparent advantages for the municipality, involvement is highly probable
	Residents	Because of the many employees and the existence of caterers and cafés in the vicinity, residents will certainly benefit from the CWS
	Local Companies	In addition to the smaller businesses, larger service providers could benefit from the workspace provided
Type of Scale of Outreach	Local (Rural) Surroundings	As can be seen from the previous point, the immediate environment is certainly touched by the CWS
	Intermediate Distance	Due to the nearby large city, the intermediate distance also comes into question as a range of influence

We successfully evaluated our taxonomy through this application to a specified environmental context. We demonstrated its validity, which *“means that the artifact works and does what it is meant to do”* (Gregor & Hevner, 2013, p. 351), and its utility. We show the latter by applying the taxonomy in a different environment from the business models of the considered CWSs, thus showing that *“the achievement of goals has value outside of the development environment”* (Gregor & Hevner, 2013, p. 351).

## Discussion

### Introducing rural coworking spaces: A new form of sustainable entrepreneurship

By providing a taxonomy of CWSs in rural areas, the paper contributes to the nascent research body. In particular, we seek to bridge the gap between the numerous diverse but in-depth papers, primarily based on urban CWSs, and the few and often more superficial papers on rural CWSs.

The operation of a CWS is, above all, a form of entrepreneurship (Bouncken et al., 2020a, b, c). From this point of view, we connect rural CWSs – a promising emerging form of sustainable entrepreneurship – to business model literature. We also introduce it to management and entrepreneurship research, as their operation requires inter alia profound management skills (Walden, 2019).

We aim to simplify the comparison of different typologies of rural CWSs – both among themselves and those observed for urban CWSs. To do so, we offer a structured approach and comprehensive but clearly defined taxonomy. Entrepreneurship is context-specific (Ferreira et al., 2015), and the proposed taxonomy affords more nuanced distinguishments in hybrid CWSs observed notably in rural areas. This allows for the specific naming, analysis, and comparison of business models of CWSs across different settings.

### Practical implications for rural coworking spaces

In contrast to urban environments, rural areas are characterized by their immediate surroundings, e.g., their closeness to nature and tranquillity. These specific conditions allow for a greater diversity of CWS business models. The presented taxonomy systematizes and highlights the various opportunities associated with the rural context. By evaluating our taxonomy, we have substantiated its usefulness for practitioners.

These are founders and managers, regional and national policymakers, and various user groups, including employees and entrepreneurs. It enables entrepreneurs to plan and implement their business model so that the environment and resources found in the specific context are utilized, integrated, and exploited in the best possible way. The primary consideration here is using existing resources – for example, vacancies, whose potential can be optimally exploited. For part-time entrepreneurs running rural CWSs, it can be a helpful tool to transition to full-time entrepreneurship. Policymakers can profit from representing the many concrete scenarios to reason for funding or putting supportive policies in place regionally. The taxonomy and its underlying suggestions can also motivate and inspire to update legislation in favor of this form of entrepreneurship. As such, it promotes and encourages spreading this form of means-driven frugal, and sustainable entrepreneurship.

### Limitations

Naturally, several limitations constrain the validity and generalizability of the results presented. Firstly, we have a limited number of interview partners based in Germany. Secondly, we only considered and included the operators’ perspective in the taxonomy’s design through the expert interviews. However, this is justified because these are the probable recipients of the taxonomy for business models of CWSs in rural areas. Nonetheless, getting a more pluralistic view, exemplarily by including international sources, will be interesting. Moreover, the presented analysis is based exclusively on qualitative data collection, limiting the elaborated findings’ generalizability.

### Conclusion and future research

The primary result of this study is the taxonomy for business models of CWSs in rural areas, represented in the structure of a morphological box. The taxonomy presents different possible forms of all parts of a business model, according to Osterwalder and Pigneur (2010) in a rural context, facilitating the creation of new business models and their implementation in practice. We demonstrated and evaluated its utility through a case study and expert interviews. In addition to the structured overview, the study’s main findings are that rural CWSs are complex entities that can take on various forms and are characterized by pronounced individuality.

Based on our findings, it would be an interesting goal to find out whether, despite the intentional individuality, archetypes or variants of rural CWS business models exist that are more successful or sustainable than others. Furthermore, an extension of the number of CWSs considered is advisable to examine the validity and completeness of the taxonomy presented. Expanding the investigations geographically and span countries and possibly continents will be sensible. This can lead to land- or region-specific archetypes or cross-country findings. The use of quantitative data is also desirable, as this study is based exclusively on qualitative data collection.

Another promising development we briefly mention in our study is organized networking among CWSs in rural areas. For a start, we identified two trends: cross-company networking, primarily via organizations such as the Germany-wide cooperative CoWorkLand, and company-internal networking through integrating several rural CWSs into an overarching umbrella brand. Furthermore, we observed a movement towards intensifying these bonds, exemplarily through the anticipated formation of (virtual) cross-CWS platforms, e.g., including a joint booking platform. Therefore, future research would undoubtedly be interesting to examine this aspect, especially in the entrepreneurial context.

## Data Availability

The interview data underlying the findings of this study are not publicly available due to restrictions on participant confidentiality. However, anonymized interview transcripts may be made available to qualified researchers upon reasonable request. Please contact the corresponding author for access to the data.

## Appendix

### A.1 Literature search process and key findings

We conducted a structured database search following the framework presented by Brocke et al. (2009). After defining the research questions and conceptualizing the different aspects of our chosen topic, we decided on a database to search for literature. We selected the database Scopus, for which the Technical University of Munich holds a license, using the following inquiry.

*“TITLE-ABS-KEY ((co*working AND “space”) OR “shared office space” OR “office as a service” OR “shared workplace”)”*

This process results in 29 papers whose content is analyzed in detail and listed in a concept-centered approach, following Webster and Watson (2002). The representation of this concept matrix can be found in Appendix A.2. The key findings from this analysis are that empirical studies outnumber conceptional methods in the literature on CWSs. Purely economic aspects in the traditional sense are not in the foreground in the viewed set of research documents. Instead, more differentiated forms of value creation and added value, both entrepreneurial and purely social, dominate. Finally, there is a clear trend in the literature to deal mainly with CWSs in urban areas compared to CWSs in rural areas.

Since the papers retrieved from this first search mostly fail to address CWSs in RAs, we carried out an additional less structured literature search iteration, specifically by seeking out publications that contain *“rural OR countryside”* in their title, abstract, or as a key.

### A.2 Concept matrix

**Table:**

Document	Type of Research		Type of Value Added			General Perspective				Spatial Context	
	Conceptional	Empirical	Monetary	Entrepreneurial	Social	Individual User	Company User	Operators (Management, Organization, Business Model)	External Phenomenon	Urban	Rural
Appel-Meulenbroek et al. (2020)		x				x					
Avdikos and Merkel (2020)	x	x		x				x		x	x
Bednár and Danko (2020)		x				x		x		x	
Blagoev et al. (2019)								x		x	
Bouncken and Reuschl (2018)	x			x	x	x					
Bouncken et al. (2020a)	x							x			
Bouncken et al. (2018)		x	x	x				x			
Bouncken et al. (2020b)		x	x	x				x			
Bouncken et al. (2020a, b, c)		x		x		x				x	
Bueno et al. (2018)		x				x					
Cabral and van Winden (2016)		x		x				x		x	
Cabral and van Winden (2020)		x						x		x	
Capdevila (2019)		x		x		x					
Clifton et al. (2022)		x		x	x	x		x		x	
Gandini and Cossu (2021)		x			x	x				x	
Ivaldi et al. (2020)		x			x			x		x	x
Josef (2017)		x					x				
Krause (2019)		x							x	x	
Leclercq-Vandelannoitte and Isaac (2016)	x						x		x		
Marchegiani and Arcese (2018)		x		x				x		x	x
Mayerhoffer (2020)	x			x					x	x	
Orel and Alonso Almeida (2019)		x		x	x	x				x	
Orel and Alonso Almeida (2019)		x			x	x		x		x	
Seo et al. (2017)		x			x	x		x		x	
Spinuzzi (2012)		x			x	x				x	
Spinuzzi et al. (2019)		x			x	x				x	
Walden (2019)		x			x	x				x	
Waters-Lynch and Potts (2017)		x		x	x	x				x	
Yang et al. (2019)	x				x			x			
**⅀**	**6**	**23**	**2**	**12**	**12**	**15**	**2**	**14**	**3**	**19**	**3**

### B.1 List of questions

#### About the interviewee

Why did you decide to found the coworking space? What was your motivation?

What is your relationship to or role in the coworking space?

Do you work in your coworking space yourself?

Is running the coworking space your main job? Or are you employed full-time alongside that?

How would you define coworking?

#### About the coworking space

When has your coworking space been founded? Since when does your coworking space exist?

What corporate form does your coworking space implement? And what does this imply or mean for the coworking space?

Has the building been vacant?

Does your coworking space have any special equipment or facilities?

Are you the only company in the building? Or do you share the building with others, and if so, with what kind of parties and how many?

Are there any external parties involved in the coworking space, or is your coworking space independent?

What role does information technology play for you? How do you use information technology? Do you use coworking space software, or are there any specific tools that you use?

How many places do you have available in your coworking space? What is the occupancy rate of your coworking space?

Has there been any entrepreneurial or the like funding at the time of the founding of the coworking space, but also since then?

#### Offered services

What service packages do you offer your users?

On what factors are the contracts based that you offer your customers?

Is the price negotiated individually, or is there a fixed price structure?

Have you already received feedback from users which has helped you transform into a permanent/ (more) successful coworking space?

Is there any tendency among the usage tariffs you offer as to which one is the most popular or which one is most commonly used so far?

What are the main revenue streams of the coworking space?

And what are the main expenses of the coworking space?

On what factors are the contracts based that you offer your customers?

#### Customer segments

What are the most important customer segments of the coworking space?

Who is the target group of your coworking space? Who uses your coworking space? What are users of the coworking space typically like?

What is the radius, and what is the scale of outreach of the coworking space in terms of customers?

Have you been able to take users with you (from an already existing community or network)?

#### Operating the coworking space

What activities are involved in operating the coworking space?

Have you had previous experiences and activities that help run this coworking space? And if so, what were they, and what did they look like?

How many employees are working in the coworking space?

What do you consider to be the most important resources for operating the coworking space?

#### Customer relationships and channels

How would you describe the customer relationship that users have with the coworking space?

Through what channels are customers able to book the services of the coworking space?

What does it mean for a user to be a member of your coworking space?

What channels do you use to communicate with your users? And conversely, which channels do your users use to get and stay in contact with you?

And through which channels do you get the most feedback (so far)?

Is it possible for users to book through your website?

Does genuine collaboration arise between the users of the coworking space?

#### Network

Has there been any mediation or assistance, or support from the municipality?

Do partnerships exist with local or (supra-)regional companies or the like? And if so, how are they manifested? And what about the municipality?

Is your coworking space a member of the Germany-wide cooperative for rural coworking spaces (CoWorkLand)?

Is there competition, and if so, what kind of competitors are there?

Is there any evidence that the coworking space is benefiting the municipality or the local community in any way?

#### About the future

Do you integrate further rural coworking spaces into your umbrella brand? And if so, what does that process look like?

When would you define a rural coworking space as successful? What constitutes a successful coworking space?

Are you planning to open a second coworking space? How do you feel about the idea, for a start?

What potential do you see in the networking of coworking spaces in rural areas.

### B.2 Search grid

Search Grid – CWSx

Search Grid Categories

*Business Model Canvas – Value Side*

**Table:**

Customer Segments
Value Propositions
Channels
Customer Relationships
Revenue Streams

*Business Model Canvas – Efficiency Side*

**Table:**

Key Resources
Key Activities
Key Partnerships
Cost Structure

*Social Stakeholder Business Model Canvas*

**Table:**

Governance
Communities
Scale of Outreach

*Background to the emergence of the Coworking Spaces*

**Table:**

Knowledge about CW
Motivation for Coworking Space in Rural Area
Previous Funding

*General prospects of Coworking Spaces in rural areas*

**Table:**

For Municipalities
For Companies
For Users

### B.3 List of interviews

**Table:**

ID	Date	Interviewee Function	Length (in Minutes)
CWS1	24.11.2020	Co-Founder and Operator of a Rural Coworking Space	49
CWS2	25.11.2020	Project Manager of a rural Coworking Space	27
CWS3	25.11.2020	Co-Founder and Operator of a rural Coworking Space	37
CWS4	04.12.2020	Co-Founder and Operator of a rural Coworking Space	37
CWS5	10.12.2020	Founder and Operator of a rural Coworking Space	29
CWS6	22.12.2020	Founder and Operator of a rural Coworking Space	28
CWS7	07.01.2021	Founder and Operator of a rural Coworking Space	27
CWS8	13.01.2021	Coworking and Community Manager	29
CWS9	19.01.2021	Co-Founder and Responsible for the areas of People, Organisational Development, and Financing	22
CWS10	08.02.2021	Co-Founder and CEO of a rural Coworking Space	23
CWS11	09.02.2021	Founder and Operator of a rural Coworking Space	23
KR1	26.11.2020	Member of the Municipality Institutions in Kranzberg	38
KR2		Politically Engaged and Involved Local Entrepreneur	
KR3	02.12.2020	Head of Industry-University Collaboration in a major IT Company, User of a Coworking Space in a Rural Area, Expert in “New Work”	27
KR4	12.01.2021	Business Relations Executive Germany and Switzerland of an IT Company with Local Headquarters in Kranzberg	13
KR5	18.02.2021	Owner of a Local Grocery and Catering Business	11
KR6	02.03.2021	Local Building Developer and Contractor	45

## Footnotes

1 We conducted a structured database search according to Brocke et al. (2009) and created a concept matrix following Webster and Watson (2002). An overview of the literature search process and the key results can be found in Appendices A.1 and A.2.

## References

[CR1] Appel-Meulenbroek R, Weijs-Perrée M, Orel M, Gauger F, Pfnür A (2020). User preferences for coworking spaces; a comparison between the Netherlands.

[CR2] Avdikos V, Merkel J (2020). Supporting open, shared and collaborative workspaces and hubs: Recent transformations and policy implications. Urban Research and Practice.

[CR3] Bähr, U., Biemann, J., Lietzau, J., & Hentschel, P., & Bertelsmann Stiftung. (Ed.). (2020). *Coworking im ländlichen Raum: Menschen, Modelle, Trends. [Coworking in rural areas: People, models, trends]*. Retrieved November 23, 2020, from https://www.bertelsmann-stiftung.de/en/publications/publication/did/coworking-im-laendlichen-raum-all

[CR4] Bailey, K. D. (1994). *Typologies and taxonomies: An introduction to classification techniques*. Sage Publications, Inc. 10.4135/9781412986397

[CR5] Bednár, P., & Danko, L. (2020). Coworking spaces as a driver of the post-fordist city: A tool for building a creative ecosystem. *European Spatial Research and Policy, 27*(1), 105–125. 10.18778/1231-1952.27.1.05

[CR6] Blagoev B, Costas J, Kärreman D (2019). ‘We are all herd animals’: Community and organizationality in coworking spaces. Organization.

[CR7] BMEL, B., CoWorkLand eG, Netzwerk Zukunftsorte e.V., neues handeln AG. (2021). *Coworking auf dem Land: Wie es gelingt und was es dafür braucht*. Retrieved July 15, 2022, from https://www.bmel.de/SharedDocs/Downloads/DE/Broschueren/coworking-land-bule.html

[CR8] Bouncken R, Reuschl AJ (2018). Coworking-spaces: How a phenomenon of the sharing economy builds a novel trend for the workplace and for entrepreneurship. Review of Managerial Science.

[CR9] Bouncken R, Laudien SM, Fredrich V, Görmar L (2018). Coopetition in coworking-spaces: Value creation and appropriation tensions in an entrepreneurial space. Review of Managerial Science.

[CR10] Bouncken R, Kraus S, Martínez-Pérez JF (2020). Entrepreneurship of an institutional field: The emergence of coworking spaces for digital business models. International Entrepreneurship and Management Journal.

[CR11] Bouncken R, Ratzmann M, Barwinski R, Kraus S (2020). Coworking spaces: Empowerment for entrepreneurship and innovation in the digital and sharing economy. Journal of Business Research.

[CR12] Bouncken, R., Qiu, Y., & Clauss, T. (2020b). Coworking-Space Business Models: Micro-Ecosystems and Platforms - Insights from China. *International Journal of Innovation and Technology Management, 17*(6). 10.1142/S0219877020500443

[CR13] Brocke, J. V., Simons, A., Niehaves, B., Riemer, K., Plattfaut, R., & Cleven, A. (2009). *Reconstructing the giant: On the importance of rigour in documenting the literature search process*. Proceedings of the European Conference on Information Systems.

[CR14] Bueno S, Rodríguez-Baltanás G, Gallego MD (2018). Coworking spaces: a new way of achieving productivity. Journal of Facilities Management.

[CR15] Cabral V, Van Winden W (2016). Coworking: An analysis of coworking strategies for interaction and innovation. International Journal of Knowledge-Based Development.

[CR16] Cabral V, Van Winden W (2020). The promise of coworking environments: A content analysis of the positioning of collaborative workspaces in Amsterdam. International Journal of Entrepreneurship and Small Business.

[CR17] Capdevila I (2019). Joining a collaborative space: Is it really a better place to work?. Journal of Business Strategy.

[CR18] Choudhury, P. R. (2020). *Our work-from-anywhere future. Best practices for all-remote organizations*. Harvard Business Review (November-December). Retrieved July 18, 2022 from https://hbr.org/2020/11/our-work-from-anywhere-future

[CR19] Clifton, N., Füzi, A., & Loudon, G. (2022). *Coworking in the digital economy: Context, motivations, and outcomes*. Futures. Advance online publication. 10.1016/j.futures.2019.102439

[CR20] Dähner, S., Reibstein, L., Slupina, M., Klingholz, R., Hennig, S., & Gruchmann, G. (2019). *Urbane Dörfer: Wie digitales Arbeiten Städter aufs Land bringen kann. [Urban Villages: How Digital Working Can Bring City Dwellers to the Countryside]* (1. Auflage ed.). Berlin: Berlin-Institut für Bevölkerung und Entwicklung and Neuland21 e.V. Retrieved July 14, 2022, from https://www.berlin-institut.org/studien-analysen/detail/urbane-doerfer

[CR21] Engstler, M., & Mörgenthaler, L. (2018). *Kreativwirtschaft im Ländlichen Raum: Kommunikationskonzept und Förderansätze: Situation und Potenziale von Coworking zur Förderung der Kreativwirtschaft im Ländlichen Raum in Baden-Würtemberg [Creative Industries in Rural Areas: Communication Concept and Funding Approaches. Situation and Potentials of Coworking for the Promotion of the Creative Industries in Rural Areas in Baden-Wuerttemberg]*. Retrieved November 3, 2020, from https://hdms.bsz-bw.de/frontdoor/index/index/docId/6430

[CR22] Ferreira, J. J., Fernandes, C., Raposo, M., Thurik, R., & Faria, J. (2015). Entrepreneur location decisions across industries. *International Entrepreneurship and Management Journal, 12*. 10.1007/s11365-015-0370-7

[CR23] Fuzi A (2015). Co-working spaces for promoting entrepreneurship in sparse regions: The case of South Wales. Regional Studies, Regional Science.

[CR24] Gandini, A., & Cossu, A. (2021) The third wave of coworking: ‘Neo-corporate’ model versus ‘resilient’ practice. *European Journal of Cultural Studies, 24*(2), 430–447. 10.1177/1367549419886060

[CR25] Gläser J, Laudel G (2010). Experteninterviews und qualitative Inhaltsanalyse als Instrumente rekonstruierender Untersuchungen [Expert interviews and qualitative content analysis as instruments of reconstructive studies] (4.

[CR26] Gregor, S., & Hevner, A. R. (2013). Positioning and Presenting Design Science Research for Maximum Impact. *MIS Quarterly, 37*(2), 337–356. 10.25300/misq/2013/37.2.01

[CR27] Gregori, P., & Holzmann, P. (2020). Digital sustainable entrepreneurship: A business model perspective on embedding digital technologies for social and environmental value creation. *Journal of Cleaner Production, 272*, 122817. 10.1016/j.jclepro.2020.122817

[CR28] Hossain, M., & Sarkar, S. (2021). Frugal Entrepreneurship: Profiting With Inclusive Growth. *IEEE Transactions on Engineering Management, PP*. 10.1109/TEM.2021.3088589

[CR29] Hummels H, Argyrou A (2021). Planetary demands: Redefining sustainable development and sustainable entrepreneurship. Journal of Cleaner Production.

[CR30] Ivaldi S, Galuppo L, Calvanese E, Scaratti G (2020). Coworking space as a practised place between welfare working and managerial challenges. Journal of Workplace Learning.

[CR31] Josef, B. (2017). *Coworking from the company’s perspective - Serendipity-biotope or getaway-spot?* BLED 2017 Proceedings. 32. Retrieved November 20, 2020, from https://aisel.aisnet.org/bled2017/32

[CR32] Joyce A, Paquin RL (2016). The triple layered business model canvas: A tool to design more sustainable business models. Journal of Cleaner Production.

[CR33] Kojo I, Nenonen S (2016). Typologies for co-working spaces in Finland – what and how?. Facilities.

[CR34] Kraus S, Bouncken RB, Görmar L, González-Serrano MH, Calabuig F (2022). Coworking spaces and makerspaces: Mapping the state of research. Journal of Innovation & Knowledge.

[CR35] Krause, I. (2019). Coworking spaces: Windows to the future of work? changes in the organizational model of work and the attitudes of the younger generation. *Foresight and STI Governance, 13*(2), 52–60. 10.17323/2500-2597.2019.2.52.60

[CR36] le Loarne S, Razgallah M, Maalaoui A, Kraus S (2022). Becoming a green entrepreneur: An advanced entrepreneurial cognition model based on a practiced-based approach. International Entrepreneurship and Management Journal.

[CR37] Leclercq-Vandelannoitte A, Isaac H (2016). The new office: How coworking changes the work concept. Journal of Business Strategy.

[CR38] Marchegiani, L., & Arcese, G. (2018). Collaborative spaces and coworking as hybrid workspaces: Friends or foes of learning and innovation? In P. Boccardelli, M. Annosi, F. Brunetta, & M. Magnusson (Eds.), *Learning and innovation in hybrid organizations*. Palgrave Macmillan, Cham. 10.1007/978-3-319-62467-9_4

[CR39] Mayerhoffer M (2020). Growth factors of the coworking industry: the case of Prague. Journal of Property Investment and Finance.

[CR40] Mediteranean, C. I. (2018). *Territorial study on coworking assets for urban and rural areas (Deliverable 3.7.1)*. Interreg Mediteranean COWORKMed. Retrieved January, 20, 2021, from https://coworkmed.interreg-med.eu/what-we-achieve/deliverable-database/detail/?tx_elibrary_pi1%5Blivrable%5D=1941&tx_elibrary_pi1%5Baction%5D=show&tx_elibrary_pi1%5Bcontroller%5D=Frontend%5CLivrable&cHash=e8f1fe9007d5787a421f0f07115bd05d

[CR41] Merten, M. (2020, 29.11.2020). *Die Menschen ziehen zurück aufs Land: Kommt die Corona-Stadtflucht? [People move back to the countryside: Is the Corona urban exodus coming?]*. Handelsblatt. Retrieved July 18, 2022, from https://www.handelsblatt.com/technik/digitale-revolution/pandemie-die-menschen-ziehen-zurueck-aufs-land-kommt-die-corona-stadtflucht/26660870.html?ticket=ST-5236695-iZcMdOrq6YnG7ZK2zRdf-ap2

[CR42] Nakano D, Shiach M, Koria M, Vasques R, Santos EGD, Virani T (2020). Coworking spaces in urban settings: Prospective roles?. Geoforum.

[CR43] Nickerson RC, Varshney U, Muntermann J (2013). A method for taxonomy development and its application in information systems. European Journal of Information Systems.

[CR44] Orel M, Alonso Almeida MM (2019). The ambience of collaboration in coworking environments. Journal of Corporate Real Estate.

[CR45] Osterwalder A, Pigneur Y (2010). Business model generation: A handbook for visionaries, game changers, and challengers.

[CR46] Prochazka, V., & Wingartz, N. (2019). *Innovation und Digitalisierung in den Kommunen und Landkreisen Baden-Württembergs: Status Quo, Herausforderungen, Bedarfe, Handlungsempfehlungen*. Frauenhofer-Institut für Arbeitswirtschaft und Organisation IAO Universität Stuttgart. Retrieved February 1, 2021, from http://publica.fraunhofer.de/dokumente/N-555265.html

[CR47] Rose, J. (2020, 06.08.2020). Time to move? Data suggests Americans may flee to rural areas post-COVID. Forbes. Retrieved July 18, 2022, from https://www.forbes.com/sites/jrose/2020/08/06/time-to-move-data-suggests-americans-may-flee-to-rural-areas-post-covid/

[CR48] Roundy PT (2019). “It takes a village” to support entrepreneurship: Intersecting economic and community dynamics in small town entrepreneurial ecosystems. International Entrepreneurship and Management Journal.

[CR49] Salwin, M., Jacyna-Gołda, I., Kraslawski, A., & Waszkiewicz, A. E. (2022). The Use of Business Model Canvas in the Design and Classification of Product-Service Systems Design Methods. *Sustainability (Switzerland), 14*(7). 10.3390/su14074283

[CR50] Seo, J., Lysiankova, L., Ock, Y. S., & Chun, D. (2017). Priorities of coworking space operation based on comparison of the hosts and users’ perspectives. *Sustainability (Switzerland), 9*(8). 10.3390/su9081494

[CR51] Spinuzzi C (2012). Working alone together: Coworking as emergent collaborative activity. Journal of Business and Technical Communication.

[CR52] Spinuzzi C, Bodrožić Z, Scaratti G, Ivaldi S (2019). “Coworking is about community”: But what is “community” in coworking?. Journal of Business and Technical Communication.

[CR53] Voll, J., Cordes, C., & Henkels, W.-N. (2021). *Coworking-Kultur im ländlichen und urbanen Raum*. Retrieved July 15, 2022, from https://www.coworking-germany.org/thesenpapier-coworking-kultur-im-laendlichen-und-urbanen-raum-veroeffentlicht/

[CR54] Walden J (2019). Communicating role expectations in a coworking office. Journal of Communication Management.

[CR55] Waters-Lynch J, Potts J (2017). The social economy of coworking spaces: A focal point model of coordination. Review of Social Economy.

[CR56] Webster, J., & Watson, R. T. (2002). Analyzing the past to prepare for the future: Writing a literature review. *MIS Quarterly, 26*(2), xiii–xxiii. Retrieved November 3, 2020, from http://www.jstor.org/stable/4132319

[CR57] Wright, D. (2018). Match made in heaven: Investment benefits of coworking spaces in historic sacred places. Retrieved January 20, 2021, from https://ecommons.cornell.edu/handle/1813/70815

[CR58] Yang E, Bisson C, Sanborn BE (2019). Coworking space as a third-fourth place: Changing models of a hybrid space in corporate real estate. Journal of Corporate Real Estate.

[CR59] Yin, R. K. (2018). *Case study research and applications: Design and methods* (6th ed.). SAGE Publications, Inc. Retrieved February 1, 2021, from https://books.google.com.eg/books?id=6DwmDwAAQBAJ

[CR60] Zinke, G., Ferdinand, J.‑P., Groß, W., Möring, J. L., Nögel, L., Petzolt, S., Richter, S., Robeck, M. S., Wessels, J. (March 2018). *Trends in der Unterstützungslandschaft von Start-ups - Inkubatoren, Akzelereatoren und andere: Studie im Auftrag des Bundesministeriums für Wirtschaft und Energie (BMWi). [Trends in the startup support landscape - incubators, accelerators and others. Study commissioned by the Federal Ministry for Economic Affairs and Energy (BMWi)]*. Germany, Berlin. Retrieved July 15, 2022, from https://www.bmwi.de/Redaktion/DE/Publikationen/Studien/trends-in-der-unterstuetzungslandschaft-von-start-ups.html

